# The Integrated System for Public Health Monitoring of West Nile Virus (ISPHM-WNV): a real-time GIS for surveillance and decision-making

**DOI:** 10.1186/1476-072X-4-21

**Published:** 2005-09-13

**Authors:** Pierre Gosselin, Germain Lebel, Sonia Rivest, Monique Douville-Fradet

**Affiliations:** 1 Institut national de santé publique du Québec (INSPQ), 945 Wolfe avenue, Sainte-Foy (Quebec), G1V5B3, Canada; 2 Centre hospitalier universitaire de Québec (CHUQ), 2705 Laurier boulevard, Sainte-Foy (Quebec), G1V 4G2, Canada; 3 Centre for Research in Geomatics, Laval University (Quebec), G1K 7P4, Canada

## Abstract

**Background:**

After its first detection in North America in New York in 1999, West Nile virus was detected for the first time in 2002 in the province of Quebec, Canada. This situation forced the Government of Quebec to adopt a public health protection plan against the virus. The plan comprises several fields of intervention including the monitoring of human cases, *Corvidae *and mosquitoes in order to ensure the early detection of the presence of the virus in a particular area. To help support the monitoring activities, the *Integrated System for Public Health Monitoring of West Nile Virus *(ISPHM-WNV) has been developed.

**Results:**

The ISPHM-WNV is a real-time geographic information system for public health surveillance of West Nile virus and includes information on *Corvidae*, mosquitoes, humans, horses, climate, and preventive larvicide interventions. It has been in operation in the province of Quebec, Canada, since May 2003. The ISPHM-WNV facilitates the collection, localization, management and analysis of monitoring data; it also allows for the display of the results of analyses on maps, tables and statistical diagrams.

**Conclusion:**

The system is very helpful for field workers in all regions of the province, as well as for central authorities. It represents the common authoritative source of data for analysis, exchange and decision-making.

## Background

He conquered Persia, Greece and Babylon and was revered and feared in East and West alike. At the age of 32, in the height of his power and glory, he was brought down... by a mosquito. According to recent speculations, Alexander the Great may have died as a result of infection by West Nile virus [1].

West Nile virus (WNV) infections may have been occurring in the Middle East for centuries. The virus has spread to new areas of the world and to new populations, causing infections that are characterized by signs and symptoms. Detected in North America for the first time in 1999 in the state of New York, it has since spread throughout the continent. Three years later, in 2002, the number of human cases of infection by this virus had increased dramatically. In the United States, 4156 cases and 284 deaths were confirmed. Moreover, 9862 confirmed cases (with 264 deaths) were reported in 2003 and 2470 (with 88 deaths) in 2004 (as of January 11^th^, 2005) [2].

The WNV was detected in mosquitoes, *Corvidae *(American Crows, Blue Jays and Common Ravens in our context) and humans for the first time in the province of Quebec, Canada, in early summer of 2002. A total of 20 confirmed human cases (including 3 deaths) were reported in 2002, 17 confirmed cases in 2003 (no deaths) and 3 cases in 2004 (1 death). This situation forced the Government of Quebec to adopt, in 2003, a public health protection plan against the virus [3] that remains in place to date. The main objectives of this plan are: to prevent complications and human deaths related to WNV infections, to ensure the early detection of the presence of the virus in a geographic area, to identify areas of potential transmission to humans for preventive action and monitoring, and to qualify the level of transmission to humans. The plan comprises several areas of intervention and their performance criteria:

- Monitoring: an integrated monitoring system (human, ornithological, entomological) in real-time;

- Laboratory: speed and provincial autonomy with regards to diagnosis (human, ornithological, entomological);

- Information: a communication plan;

- Intervention: rapid, effective, and flexible to adjust to the evolution of the epidemiologic situation;

- Research and evaluation: of the effectiveness and impacts of the actions taken;

- Decision process: a public health structure to optimize intervention capacity.

Given the particular epidemiologic character of the infection (avian amplifiers, transmission by mosquito vectors), the monitoring component of the plan is comprised of three interrelated elements:

- The monitoring of human cases of infection: the presence of infected persons that have acquired the infection locally confirms an active transmission of the WNV in the concerned area;

- The monitoring of animals: the presence of clustered dead *Corvidae *infected by the WNV indicates a potential source of amplification; these observations help identify target sites for mosquito monitoring;

- The monitoring of mosquitoes: the presence of a pool of infected mosquitoes indicates that a WNV source exists that presents a potential for the transmission of WNV to humans.

Analysis of the monitoring data makes it possible to target preventive interventions such that the appropriate personal, community, and environmental protection interventions can be considered; however, to be useful, the monitoring data must be available in real-time. The development of an information system to support the monitoring component of the intervention plan for the province of Quebec was entrusted to the *Institut national de santé publique du Québec *(INSPQ) in 2003 by the *Ministère de la Santé et des Services sociaux *(MSSS). The mandate of the INSPQ includes the implementation and continual update of an extranet web site dedicated to online data entry, data warehousing and document exchange. Moreover, this site included tables or graphs of relevant surveillance data, an online tool ensuring the validation and geographic localization of relevant events and an online real-time mapping tool for the integration of all data. The INSPQ thus developed the *Integrated System for Public Health Monitoring of West Nile Virus *(ISPHM-WNV) that allows the different actors involved to rapidly and easily assess the situation and recommend adapted interventions.

Using this system, the evaluation of the epidemiologic situation is carried out by an Expert Group that includes representatives of the appropriate government departments, scientists, and regional health authorities, in order to recommend the optimal interventions against WNV. In the event of a major epidemic situation, a high-level Advisory Committee is consulted on interventions before the Deputy Minister makes a decision.

In Canada, several provincial surveillance systems that include a WNV monitoring component have been implemented in the last few years, and information is shared through the Canadian Network for Public Health Intelligence (CNPHI). The CNPHI is targeted at improving the capacity of the Canadian health system to reduce human illness associated with infectious disease events by supporting intelligence exchange, surveillance activities and outbreak investigations. It is a national framework to collect and process surveillance data, disseminate strategic intelligence, and coordinate response to biological threats [4]. It comprises the West Nile virus Monitor, a surveillance component fed with provincial monitoring data that allows the visualisation of maps and tables. The provinces are responsible for the monitoring of the virus on their respective territory and the implementation of programs and systems to support this task. For example, the British Columbia Centre for Disease Control has implemented the Internet Geographic Information System (GIS) that allows the interactive mapping of various monitoring data related to WNV [5]. In the United States, the Centers for Disease Control and Prevention (CDC) have implemented the National WNV Surveillance System [6]. The objectives of the system are to monitor the geographic and temporal spread of WNV, develop national public health strategies for WNV surveillance, prevention, and control, develop a more complete regional picture of the geographic distribution and incidence of the other clinically important arboviruses, and provide national and regional information to public health officials, elected government officials, and the public. The data entry is done by reporting jurisdictions at the state level through the ArboNET system, the national electronic surveillance system established by CDC to assist states in tracking WNV and other mosquito-borne viruses. The system includes a mapping component and the maps it produces are available on the United States Geological Survey (USGS) web site. On a state or local level, WNV monitoring programs and infrastructures have been implemented. For example, see [7] for information on the infrastructure implemented by the State of New-York. The City of New York has also implemented the Dynamic Continuous-Area Space-Time (DYCAST) GIS system that was developed to identify and prospectively monitor high-risk areas for WNV [8].

In line with those previous examples, this paper outlines the context, architecture and different capabilities of the ISPHM-WNV, including its spatial and cartographic functionalities. In addition, examples of its utility as a tool for the management of risks and the reporting of results on a daily basis are presented. Finally, the paper explores future considerations for the development of such systems.

## Results

### The Integrated System for Public Health Monitoring of West Nile Virus (ISPHM-WNV)

Various data collection methods have been organized and implemented in order to feed the ISPHM-WNV. The 18 regional health authorities of the province are responsible for the mandatory reporting of all human cases of infection by WNV. The population is invited, by means of media campaigns, to report the presence of dead *Corvidae *to a reporting center (WNV-Info line). The location of reported *Corvidae *must be precisely indicated to allow for their collection and subsequent testing. Mosquito monitoring is performed at fixed monitoring stations as well as in potential risk zones identified following the analysis of infected bird clusters.

It is essential that the ISPHM-WNV support the integration of the different data sources and provide the required functionalities to assist the numerous actors with their respective tasks (data entry, data localization, data validation, data visualization, data analysis and decision-making).

### The context and the architecture of the ISPHM-WNV

The INSPQ is responsible for:

- The implementation and the continual update, on a daily basis, of a secured extranet web site that is used to allow the exchange of documents and to update the monitoring database;

- The development and deployment, on the extranet network, of an on-line tool for the validation and the geographic localization of the various types of events recorded in the database;

- The development and deployment of a cartographic tool that provides an on-line cartographic representation of the monitoring data;

- The development and the update of charts and graphics of the temporal frequency distributions of the various events recorded in the database;

- The creation of an Expert Group to support the Advisory Committee in the interpretation of the results and in the analysis and the detection of aggregates, in time and space, of reported bird cases, of samples of infected mosquitoes, and of suspected and confirmed human cases.

A team of six people was formed and ensured the development and the management of the ISPHM-WNV. Moreover, the Expert Group was created to assist the development of a systematic analysis protocol for the data contained in the ISPHM-WNV, propose criteria for alerts, increase or withdraw surveillance components, produce the basic relevant analyses (at the provincial and regional health authority levels), detect significant sources of WNV activity, interpret the results, adjust interventions and promptly inform the Advisory Committee if significant expansion of insecticide use is needed. The committee also provides relevant technical opinions in order to improve the monitoring of WNV and the ISPHM-WNV itself.

**Figure 1:**
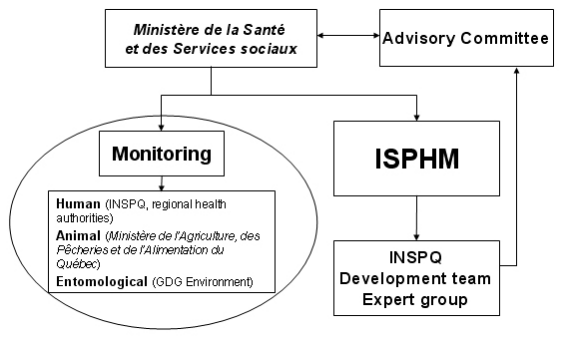
The main organizations and groups involved in the Integrated System for Public Health Monitoring of West Nile Virus (ISPHM-WNV).

The context of the system is shown in Figure 1.

The extranet site of the ISPHM-WNV constitutes a portal to the different monitoring data collected, and is therefore principally intended for use by the regional public health professionals of the province. The integrated data presented on the extranet site of the ISPHM-WNV are divided into four categories: descriptions and analyses of dead *Corvidae*, and analyses of mosquitoes, human cases, and animals (horses and others wild animals tested).

The INSPQ purchased an internet-based cartographic representation software and consultation services for the development of a customized georeferencing tool for the geographic localization of the various data. The firm KHEOPS Technologies, with its cartographic software *JMap*^® ^[9], was chosen to help fulfill the geographic processing needs of the system.

**Figure 2:**
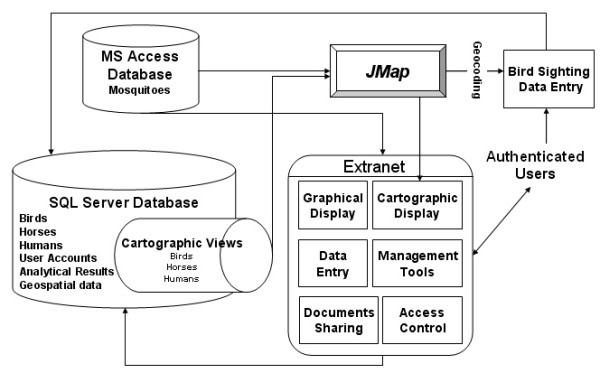
The general architecture of the Integrated System for Public Health Monitoring of West Nile Virus (ISPHM-WNV).

The main implementation steps of the first version of the ISPHM-WNV were carried out in four months in 2003:

- Opening of the reporting center (WNV-Info line) for dead *Corvidae*, including the implementation of the input forms and the tool for the geographic localization of the reports;

- First implementation of the cartographic representation tool on the extranet site;

- Implementation of a complete version of the cartographic representation tool;

- Implementation of the georeferencing tool for the batch geographic localization of events;

Since then, continuous improvements have been made to the system. The general architecture of the ISPHM-WNV is presented in Figure 2[10]. The different components will be described in more detail in the following sections.

### The main components of the ISPHM-WNV – database

#### MS^® ^SQL Server database

This database supports the entire system and contains not only the monitoring data but also all the data required for the proper functioning and maintenance of the system, including user accounts information, geographic data, the different analysis results and the shared documents.

### The main components of the ISPHM-WNV – JMap

#### Cartographic functionalities

*JMap*^® ^is an open solution for publishing interactive spatial applications over enterprise, public, and mobile networks [9]. The main characteristics and functionalities of the software are:

- On-line access (Java applets);

- Remote on-line administration via a secured web site;

- Integration of cartographic data of different formats;

- Unlimited number of simultaneous users;

- Cartographic navigation tools and measurement tools;

- Spatial analysis functions;

- Construction of different types of thematic maps;

- Flexible selection of information for display (time periods or qualitative attributes);

- Dynamic links of geographical locations to multimedia documents;

- On-line collaboration (red-lining) and email exchange of annotated information;

- Export data to common office software.

#### Georeferencing tool

A georeferencing tool based on the street addresses has been developed in JMap^® ^and integrated to the ISPHM-WNV. This tool allows for the precise localization of dead bird reports and infection cases. The base road segments database used is CanMap Streetfiles from DMTI Spatial [11]. The tool includes a phonetic matching algorithm to help correct typographic errors that can occur during manual data entry.

Two versions of the georeferencing tool allow for individual localization of bird reports (for the telephonic reporting center) or batch processing for animal and human cases.

### The main components of the ISPHM-WNV – extranet site

The extranet site includes:

- A *News *section containing the latest scientific information available on the WNV;

- An *Overview *section allowing for rapid assessment of the situation;

- A *Rapid links *section containing links to statistical information related to the different types of monitoring;

- A *Shared documents *section allowing the different users to exchange documents of any type;

- A discussion forum;

- Links to the different monitoring statistics (tables and graphs) sections of the system: human cases, *Corvidae*, mosquitoes and other animals;

- A link to the cartographic display software (JMap^®^) allowing for the visualization and the spatial analysis of the monitoring data;

- Links to the administration sections.

#### Monitoring data – human cases

This component of the system allows the *Laboratoire de santé publique du Québec *and the representatives of the various regional health authorities to manually enter, validate and visualize information related to human cases of WNV infection. The cases are localized using the georeferencing tool described earlier for representation on a map, along with other pertinent information.

#### Monitoring data – Corvidae reports

This component of the system allows the operators of the reporting center to manually enter, validate, and visualize the information related to reported dead *Corvidae*. When they become available, the laboratory tests' results are entered by the laboratory staff. The georeferencing tool allows the operators to immediately validate and geographically position the discovery site and automatically identify and contact the right person to collect the dead bird for laboratory tests. An algorithm was also implemented to help the telephone operator localize the birds when a civic address is not available. Spatial analysis is also performed automatically on each localized bird to determine if collection of the specimen is necessary, according to the number of positive birds already found in the neighbourhood.

#### Monitoring data – mosquitoes

This component of the system presents a control panel allowing for the transfer of laboratory results and for the calculation of infection rates of the monitored mosquito pools. Statistics are also available on temporal and regional counts of mosquitoes by species, and also on positive pools upon which infection rates are automatically computed using a software developed at the Center for Ecological Entomology, Illinois Natural History Survey (Gu Weidong, personal communication, 2003).

#### Monitoring data – other animals

This component of the system allows representatives of the Québec *Ministère de l'Agriculture, des Pêcheries et de l'Alimentation *(MAPAQ) to manually enter, validate, and visualize information related to infection cases in horses and other animals as necessary.

#### Graphical display – statistics

**Figure 3:**
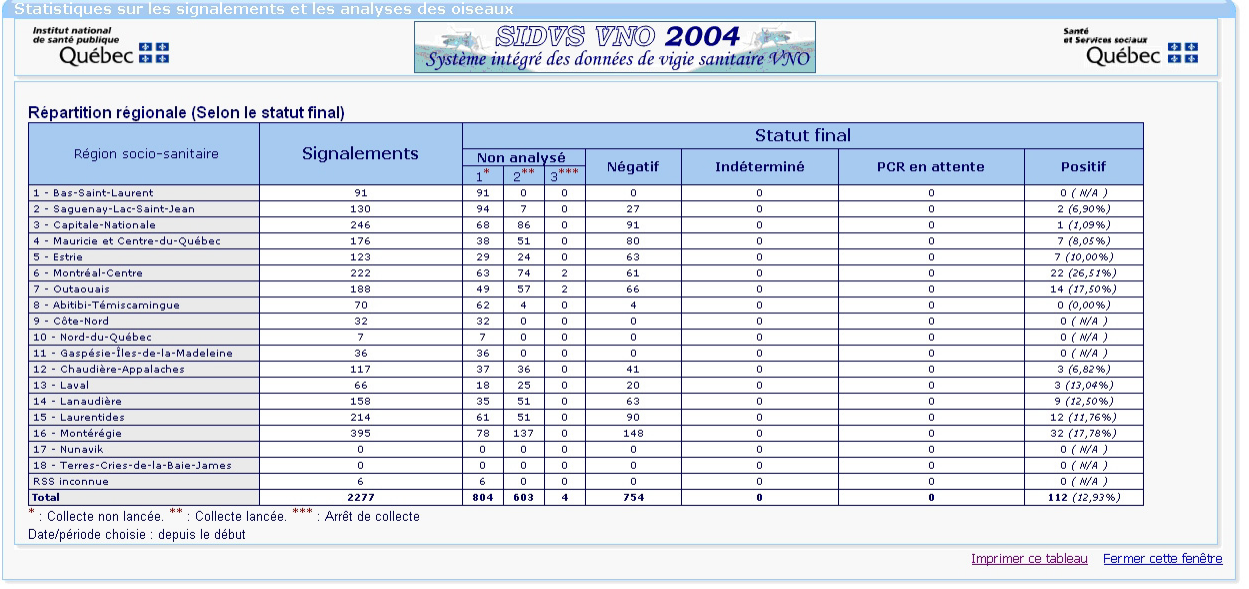
A tabular display example showing the statistics related to dead bird reporting per region in 2004.

This component contains, for each monitoring domain, tables and graphics for the spatial (provincial and regional) and temporal distribution of monitoring data. The main statistics page presents the information at the provincial level. The users can then select a particular region for more specific analyses. It is also possible to perform custom analyses for any specified time period. Figure 3 presents an example of a statistical table summarizing information related to the reporting and laboratory tests on dead *Corvidae*, per region. It comprises a first column containing the regions, a second column containing the number of reports and a series of columns containing the status of the laboratory tests (not analyzed, negative, undetermined, pending or positive).

**Figure 4:**
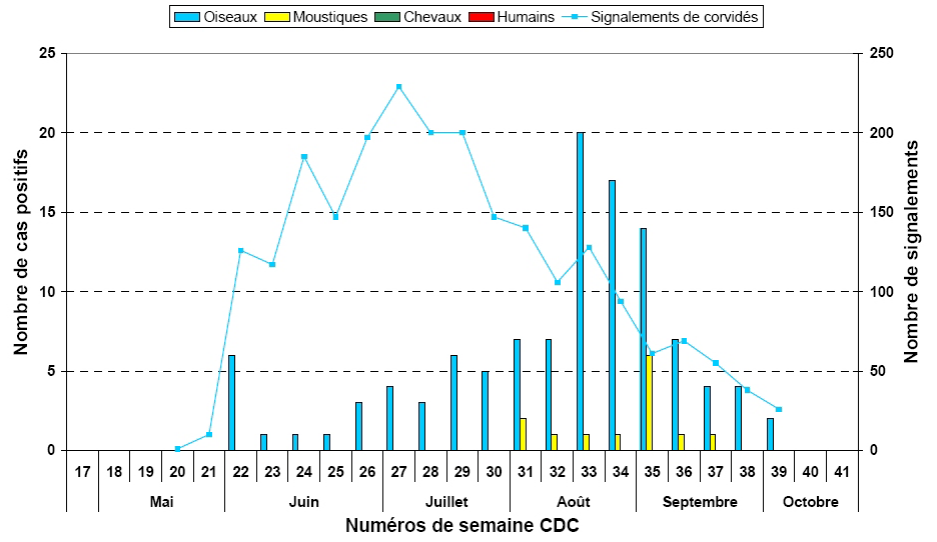
**Bar chart example presenting the number of dead *Corvidae *reported and the number of positive cases of infection for the year 2004**. This bar chart presents the number of dead *Corvidae *reported (blue line) and the number of positive cases of infection for the year 2004 for birds (blue bars) and mosquitoes (yellow bars). No cases of horses or humans were reported.

Figure 4 presents an example of a chart representing the number of dead *Corvidae *reported (blue line) and the number of positive cases of infection for the year 2004 for birds (blue bars) and mosquitoes (yellow bars). There were no human or horse cases in 2004.

#### Cartographic display

This component of the ISPHM-WNV contains a cartographic interface allowing for the visualization and analysis of thematic maps including different layers of information that can be displayed according to specific user needs:

- Limits of the regional health authorities;

- Limits of the local health authorities;

- Administrative regions;

- Regional county municipalities;

- Cities and boroughs;

- Population density by dissemination (statistical) area;

- Topography;

- Hydrography;

- Vegetation zones;

- Road network;

- Rail network;

- Hospitals;

- National parks;

- Bird reports (for previous years and year-to-date, with laboratory tests results);

- Infected *Corvidae *(for previous years and year-to-date);

- Crow principal roost areas in late summer;

- Mosquito pools (for previous years and year-to-date, with laboratory tests results);

- Infected mosquito pools (for previous years and year-to-date);

- Mosquito monitoring stations;

- Human cases of infection (for previous years and year-to-date);

- Equine cases (for previous years and year-to-date);

- Other infected animals (for previous years and year-to-date);

- Larvicide and insecticide sprayed areas (for previous years and year-to-date);

- Planned larvicide and insecticide treatment areas (for previous years and year-to-date);

- Meteorological data.

The different maps can present individual punctual localization of events, or aggregated data according to the different territorial divisions (ex. regional health authorities). A specific interface has been developed to allow the automatic detection of spatio-temporal clusters of *Corvidae *deaths. This interface uses the SaTScan™ freeware [12] and provides a cartographic representation of the clusters found by SaTScan™. Specific tools have also been implemented in order to filter the data shown on the maps. For all relevant surveillance data, users can also select and download data in MS^® ^Excel on their own workstation.

**Figure 5:**
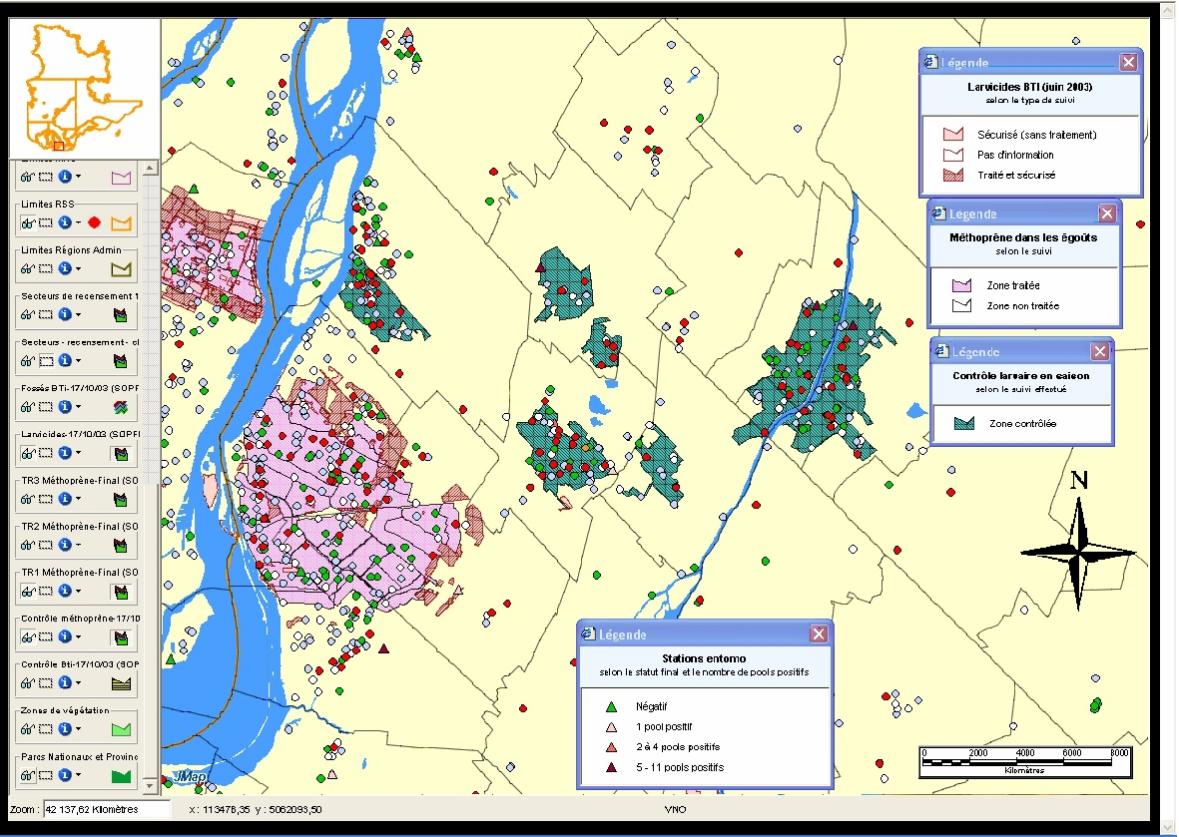
**Example of a thematic map showing the different insecticide treatments and the localization of bird reports and mosquito pools, in 2003**. This thematic map shows the different insecticide treatments (shaded areas) and the localization of bird reports (colored dots) and mosquito pools (colored triangles) along with their status, in 2003.

**Figure 6:**
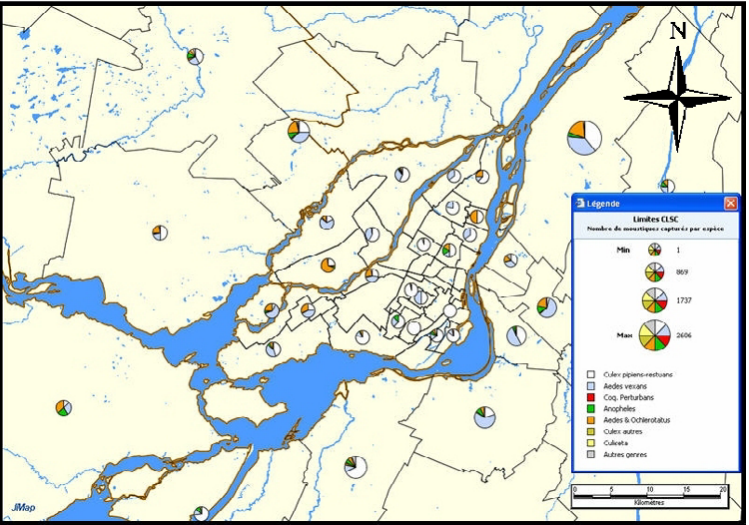
**Example of a thematic map showing the status of reported dead *Corvidae *around the Montreal Island, in 2005**. This thematic map shows the status (represented by different colors) of reported dead *Corvidae *for different territorial subdivisions around the Montreal Island, in 2005.

Figures 5 and 6 present examples of cartographic displays showing the different insecticide treatments and the localization of reported dead *Corvidae *and mosquito pools (along with their status), and the status of reported dead *Corvidae *by territory for the local health authorities around the Montreal Island, respectively. In Figure 5, the coral-shaded regions represent zones where the larvicide BTI has been used, or is planned to be used (white = no available information; light coral = checked area without treatment; dark coral = checked and treated area). The pink-shaded regions represent zones where Methoprene has been used, or is planned to be used (white = area not treated; pink = treated area). The green regions represent zones where larval control has taken place. The colored dots represent *Corvidae *reports (green = negative; white = not collected; grey = collected but not analyzed; red = positive; yellow = undetermined; purple = results pending). The colored triangles represent entomological stations (green = negative; pink = 1 positive pool; red = 2 to 4 positive pools; dark red = 5 to 11 positive pools).

In Figure 6, the pie charts represent test results of collected dead *Corvidae *(white = not collected; light blue = collected but not analyzed; grey = collection not necessary; red = positive; green = negative; purple = results pending; yellow = undetermined).

#### Management tools

This component of the system contains different tools for the management of the system. It allows the administrator to control user access to the various components of the system, including access to nominative and confidential data. It also compiles utilization statistics (number of accesses per page) and a log of all operations done by any user on the extranet website. These statistics are used to improve the ergonomics and functionalities of the system.

### Use of the ISPHM-WNV for daily management

During the active season of the virus, the Expert Group is responsible for the daily assessment of the situation and meets regularly every week to propose interventions to regional authorities and the Ministry of Health, as necessary. The Advisory Committee only meets to assess major epidemic situations and recommend the needed interventions to the public health authorities.

Interventions are grouped in four categories based on virus activity over the previous three weeks:

- Absence of activity;

- Possible source of infection (infected *Corvidae *found);

- Probable source of infection (infected mosquitoes or both infected *Corvidae *and infected mosquitoes found);

- Confirmed source of infection (human cases detected).

A multi-criteria analysis grid is used to plan the different actions to be taken (Table 1).

**Table 1:** Summary of the 2004 analysis grid containing the possible actions according to the degree of virus activity.

**Situation**	**Possible actions**
No virus activity	- Verify the dead *Corvidae *reports - Maintain preventive activities (media)

Possible source of infection (infected *Corvidae *found)	- Target the geographical zone of activity and determine the population density - Assess clusters of dead *Corvidae* - Plan for mobile mosquito monitoring - Closely monitor equine cases - Maintain preventive activities (media)

Probable source of infection (infected mosquitoes or both infected *Corvidae *and infected mosquitoes found)	- Target the geographical zone of activity and determine the population density - Prioritise the analysis of dead *Corvidae *in the affected region - Assess clusters of dead *Corvidae* - Assess mosquito pool information (species and positivity) - Closely monitor equine cases - Integrate meteorological data into the analysis - Consider insecticide treatments - Increase preventive activities (media)

Confirmed source of infection (human cases detected)	- Associate probabilities with potential acquisition site - Target the geographical zone of activity and determine the population density - Prioritise the analysis of dead *Corvidae *in the affected region - Assess clusters of dead *Corvidae* - Assess mosquito pool information (species and positivity) - Closely monitor equine cases - Integrate meteorological data into the analysis - Consider insecticide treatments - Increase preventive activities (media)

Results of the monitoring analyses are shared with the public. Every week, a map showing the current situation is produced and is published in the *Flash-VNO *bulletin by the INSPQ and disseminated to partner organizations and the media. This bulletin is also available within the WNV section of the INSPQ web site for public consultation. The data is also shared within Canada through the Canadian Network for Public Health Intelligence (CMPHI) and also appears in national summaries and websites. This can be especially useful in time of epidemic outbreak and for comparative evaluations.

### Evaluation of the ISPHM-WNV

After each active season, an evaluation of the ISPHM-WNV is performed to collect and compile the comments of users with respect to the current version of the system and to collect suggestions for system improvements in order to prepare for the future season. A first formal evaluation based on theoretical models was conducted by interviews in 2003 [13]. 86% of the respondents found that the system was easy to use. All respondents noted that the system is very useful for monitoring of the epidemiologic situation, mainly for entomological, ornithological and human surveillance. 93% of the respondents planned to use the system in the following year (2004). According to the respondents' comments, the system provides many outcomes: it facilitates and speeds data access, it allows for simplified data analysis and for the precise planning of the control interventions, and it facilitates the sharing of information among the different actors [13]. As such, the system contributes to the main objectives of the public health protection plan adopted by the Government of Quebec [3].

Some identified limitations of the 2003 version of the system were the absence of an on-line help document, the absence of a process to notify the users of a significant update (ex. a confirmed human case), the difficulties in extracting data from the system, and the slow display speed for certain types of statistical tables and diagrams. Some of these limitations have already been addressed and others will be addressed in future versions of the system. The evaluation is now based on a simpler 13-question electronic questionnaire.

For 2004, a summary of the use of the system was produced using the access control module. 155 user accounts have been created for public health specialists of the MSSS, the INSPQ, the regional health authorities, and for external partners (ex. Health Canada specialists). 76% of the user accounts have been used at least once during the season. More than 58000 web pages have been accessed from May 30^th ^to November 1^st^. The most frequently accessed section of the Extranet site was the *Corvidae *reports monitoring section, followed by the mosquito pools monitoring section and then by the cartographic interface. The cartographic tool was used at least once by 77% of the users.

On the data side, the system managed 2277 bird reports, 866 of which were analyzed. 112 of these reports were positive. Data about 8452 mosquito pools, 21 of which were positive, were also managed. To complete the statistics, 3 confirmed human cases (0 probable cases) and 0 infected horses (2 other infected animals) occurred.

### The future of the ISPHM-WNV

#### Spatial on-line analytical processing (SOLAP)

The system will be enriched, in 2005, with spatial on-line analytical processing (SOLAP) capabilities. A SOLAP tool can be defined as a software that allows rapid and easy navigation within spatial databases and that offers many levels of information granularity, many themes, many epochs and many display modes, synchronized or not: maps, tables and diagrams [14]. SOLAP tools are based on multidimensional analysis. This characteristic allows for rapid and easy analyses of data according to multiple criteria (spatial, temporal and other attributes). SOLAP tools are currently in use in the public health field [15] and their advantages are numerous:

- They aim at supporting, transparently, the way humans think and analyze;

- They have a user interface that hides the complexity of query languages;

- They allow the users to focus on the results of the navigation rather than on the analysis process itself (i.e. focus on "what to obtain" rather than on "how to obtain it");

- Their response time is practically instantaneous;

- They incorporate the cartographic capabilities that most users cannot easily access otherwise.

The multidimensional analysis capabilities will involve a particular data structure that will be updated in real-time as new monitoring data become available.

#### Future research directions

A research project is currently conducted to incorporate predictive modeling in the ISPHM-WNV. The project is based on the Multi-Agent Geo-Simulation approach and aims at simulating the behaviours of mosquitoes and *Corvidae *that are linked to the spread and transmission of WNV. This simulation is expected to take place in a virtual mapping environment representing a large territory (the province of Quebec) and according to various climate scenarios and larvicide treatments. A preliminary study carried out in 2004 determined the feasibility of the project [16], and a functional prototype is now running. It will be tested and calibrated with the collaboration of other Canadian jurisdictions in 2005–2006.

## Conclusion

This paper presents the *Integrated System for Public Health Monitoring of West Nile Virus *(ISPHM-WNV), its context, its architecture and its various functionalities as well as examples of its outputs. The ISPHM-WNV is an excellent example of integration of new technologies within the public health field.

This system rapidly became the essential tool for monitoring the activity of the West Nile virus in the province of Quebec. The system is very useful for field workers in all regions of the province, as well as for central authorities [10]. It speeds up the delivery of relevant information to all actors and simplifies the task of data analysis. It represents the common authoritative source of data for analysis, for exchange and for decision-making within the province. The INSPQ is now running the system for the 2005 season, planning to include spatial on-line analytical processing (SOLAP) functionalities to improve the facility and speed of analysis. This first successful implementation in Quebec of a real-time information system on a notifiable disease has had other positive outcomes, including the development of a similar system for other notifiable health events resulting from chemical exposures and other infectious diseases.

We believe our approach can be useful for other jurisdictions planning to implement or improve similar systems, either in Canada or elsewhere. Our system uses recent commercially available technologies and is being further developed to integrate emerging ones, such as spatial OLAP tools and other more recent approaches as they become available. Because of these characteristics, an implementation within another organisation would be done rapidly and the risks would be minimized.

## List of abbreviations

BTI: *Bacillus thuringiensis israelensis*

CDC: Centers for Disease Control and Prevention

CHUQ: *Centre hospitalier universitaire de Québec*

CNPHI: Canadian network for public health intelligence

DYCAST: Dynamic Continuous-Area Space-Time

GIS: Geographic Information System

INSPQ: *Institut national de santé publique du Québec*

ISPHM-WNV: Integrated system for public health monitoring of West Nile virus

MAPAQ: *Ministère de l'Agriculture, des Pêcheries et de l'Alimentation du Québec*

MS: Microsoft

MSSS: *Ministère de la Santé et des Services sociaux*

SOLAP: Spatial on-line analytical processing

USGS: United States Geological Survey

WNV: West Nile virus

## Authors' contributions

PG: Member of the Expert Group and the Advisory Committee in 2003, he was involved in the development and implementation of the system, and in the decision-making process. He participated in the writing and revision of the paper.

GL: He was in charge of the development and implementation of the system, and involved in the production of surveillance reports. Current member of the Expert Group and the Advisory Committee since 2003. He revised the paper.

SR: She was involved in the development of SOLAP and was responsible for the writing of the paper.

MDF: She was involved in the development of the system and has been in charge of the Expert Group and a member of the Advisory Committee since 2003 until now. She revised the paper.
